# The beneficial androgenic action of steroidal aromatase inactivators in estrogen-dependent breast cancer after failure of nonsteroidal drugs

**DOI:** 10.1038/s41419-019-1724-9

**Published:** 2019-06-24

**Authors:** Lanyang Gao, Zheng Bao, Heng Deng, Xiaofang Li, Jiamin Li, Zuyuan Rong, Youzhe Yang, Ling Liu, Dan Nie, Guilin Wang, Alexander T. Teichmann, F. Heinrich Wieland

**Affiliations:** 1 Sichuan Provincial Center for Gynaecology and Breast Disease, The Affiliated Hospital of Southwest Medical University, Southwest Medical University, Luzhou, China; 2 Safety Evalution Center, Sichuan Institute for Food and Drug Control, Chengdu, Sichuan Province China

**Keywords:** Breast cancer, Chromosome condensation, Breast cancer

## Abstract

Direct treatment of ER (+) breast cancer with Formestane diminishes the tumor within weeks. This is unlikely due to lack of estrogens alone. We proposed that it is the negative influence of androgens on the growth of ER(+) breast cancer. We investigated the influence of Formestane and Exemestane and of their major androgenic metabolites 4-hydroxytestosterone and 17-hydroexemestane on the proliferation of MCF-7 cells and ZR-75-1 cells. Inhibitory effects could be prevented by antiandrogens and siRNA. Activation of the AR in MCF-7 and U2-OS cells was tested by reporter gene assays. In vivo androgenicity was evaluated using the Hershberger assay. Influence on the cell cycle was demonstrated by flow-cytometry. Influence of androgens on the activity of *CCND1* was demonstrated by Chip-qPCR. Antitumor activity was determined by topical treatment of DMBA tumors. We found that breast cancer cells can metabolize Formestane and Exemestane to androgenic compounds which inhibit proliferation. This can be explained by hindering the accessibility of *CCND1* by histone modification. Androgenic metabolites can abolish the growth of DMBA-tumors and prevent the appearance of new tumors. The lack of cross-resistance between steroidal and nonsteroidal aromatase inhibitors is due to inhibitory effects of androgenic steroidal metabolites on the production of cyclin D1. These sterols not only inhibit proliferation of cancer cells but can also stop the growth of DMBA cancers upon direct absorption into the tumor. The quick and considerable effect on ER(+) tumors may open a new avenue for neodjuvant treatment.

## Introduction

More than 50% of breast cancers express estrogen receptor alpha (ERα) and are dependent on estrogens for their development and growth^1,2^. Consequently the major treatment strategy is deprivation of estradiol (E_2_). In the past the main method of inhibiting the cancer growth was to block ERα by tamoxifen^3^. Today in postmenopausal women estrogen deprivation is achieved by inhibiting aromatase the key enzyme of estrogen synthesis from adrenal precursor molecules^4^. Aromatase inhibitors are either steroidal or nonsteroidal. The latter are more effective in lowering systemic production of E2. Exemestane is the only steroidal inhibitor on the market which can be given by the oral route. Today it is predominantly used in combination with Everolimus, an inhibitor of mammalian target of rapamycin in advanced HER2/neu negative ER(+) breast cancer^5^.

The first specific steroidal AI in clinical use was 4-hydroxyandrostenedione (4-OHA)^6^, named Formestane. It came to the market as an intramuscular depot preparation (Lentaron^®^Depot). Lentaron^®^ is administered at a single dose of 250 mg/patient/application once every 2 weeks. Since this tedious way of administration is not suited for adjuvant treatment, Lentaron was taken from the market. Nonsteroidal AIs are chemicals which are not subjected to much metabolic changes within cells, whereas the structure of the steroidal aromatase inhibitors can undergo slight changes within cells giving rise to different active sterols (intracrinology)^7^.

Both steroidal AIs do not only inhibit the enzyme competitively but they also inactivate it by binding covalently to the active center^8^. The two steroidal AIs can be still effective, when the tumor starts to regrow despite the complete absence of E_2_ caused by nonsteroidal AIs^9–11^. In such cases the clinical benefit rate usually is around 50%, the benefit being almost exclusively disease stabilization for more than 6 months. Their clinical effects must be due to a mechanism different from deprivation of E_2_.

The preoperative treatment of breast cancers consists mainly in cytotoxic therapy of ER-negative (ER(−)) cancers^12^. ER(+) cancers are not very sensitive to cytotoxic drugs^13^ and the hormonal treatment with AIs is only moderately effective and takes at least 4 months^14^. In this view, it is very surprising that it is possible to reduce the size of luminal A tumors within 40 days by more than 50% by daily applying Formestane topically to the affected breast.

This downsizing clearly is not solely due to deprivation of E_2_, since Letrozole is just doing that and works much slower. The most likely explanation for the additional mechanism of action of steroidal AIs is androgenic activity of Formestane or Exemestane either directly or indirectly. The indirect effect would be the formation of androgenic metabolites of these drugs. It is well known, that their 17-OH derivatives (4-hydroxytestosterone: 4-OHT, and 17-hydroexemestane: 17-HEXE) have a high affinity to the AR^15,16^. It has also been described that androgenic substances can inhibit the proliferation of ER(+)/AR(+) cell lines^17–19^.

There are some indications that this inhibition is due to the down-regulation of Cyclin D1^20^. In MCF-7 cells, The AR corepressor DAX1 is recruited on the androgen-responsive region of the cyclin D1 promoter upon DHT stimulation. But it is unclear how this repressor complex modify chromatin conformation in the cyclin D1 promoter. In the present study, the results of chromatin immunoprecipitation show that androgen-activated AR decrease H3K4me3 and H3K9ac of the cyclin D1 promoter, but increase the H3K9me3. Cyclin D1 mRNA and protein are overexpressed in ~50% of primary breast cancer cases^21^.

In order to gain more information about the androgenic properties of Formestane, 4-OHT, Exemestane, and 17-HEXE, and about their intracellular metabolism and influence on certain cellular behaviors we have investigated the affinity of the sterols to the AR by competitive receptor-binding assays and their ability to activate the AR by reporter gene assays. We tried to prove that the influence on the proliferation of breast cancer cells is due to activation of the AR and that the activated AR slows down the cell cycle by influencing the expression of the Cyclin D1 gene via histone modification. We investigated the androgenic and myotrophic properties of the substances in castrated young male rats and compared the influence of different sterols on the growth of E_2_-dependent DMBA-induced breast cancers in ovariectomized rats under hormone replacement therapy in order to characterize the induction of tumor atrophy and apoptosis at physiological levels of estradiol.

## Results

### Expression of the AR in breast cancer cell lines

**Fig. 1:**
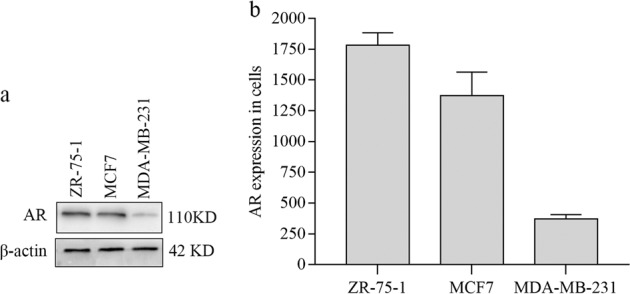
AR expression in breast cancer cell-lines. **a** Blot shows the expression of AR protein. Experimental protocol is as described in the “Materials and methods”. Blots were also probed for β-actin (bottom) to verify equal amounts of protein loaded in each lane. The ER(+) cells (MCF-7 and ZR-75-1) are expressing the AR. The ER(−) MDA-MB-231 almost have no AR. **b** The densitometric evaluations are calculated by ImageJ. These data are representative of three separate experiments, each in triplicate; bars, SEM

The presence or absence of the AR in cells was demonstrated by western-blot (Fig. 1a). Whole-cell lysates from all cell lines were used. MCF-7 and ZR-75-1 cells are breast cancer cells expressing ERα and AR (Fig. 1a). MDA-MB-231 cells were weakly stained for AR, which indicated that this cell line was AR negative. These results were consistent with the densitometric analysis (Fig. 1b).

### Influence of tested compounds on the proliferation of breast cancer

**Fig. 2:**
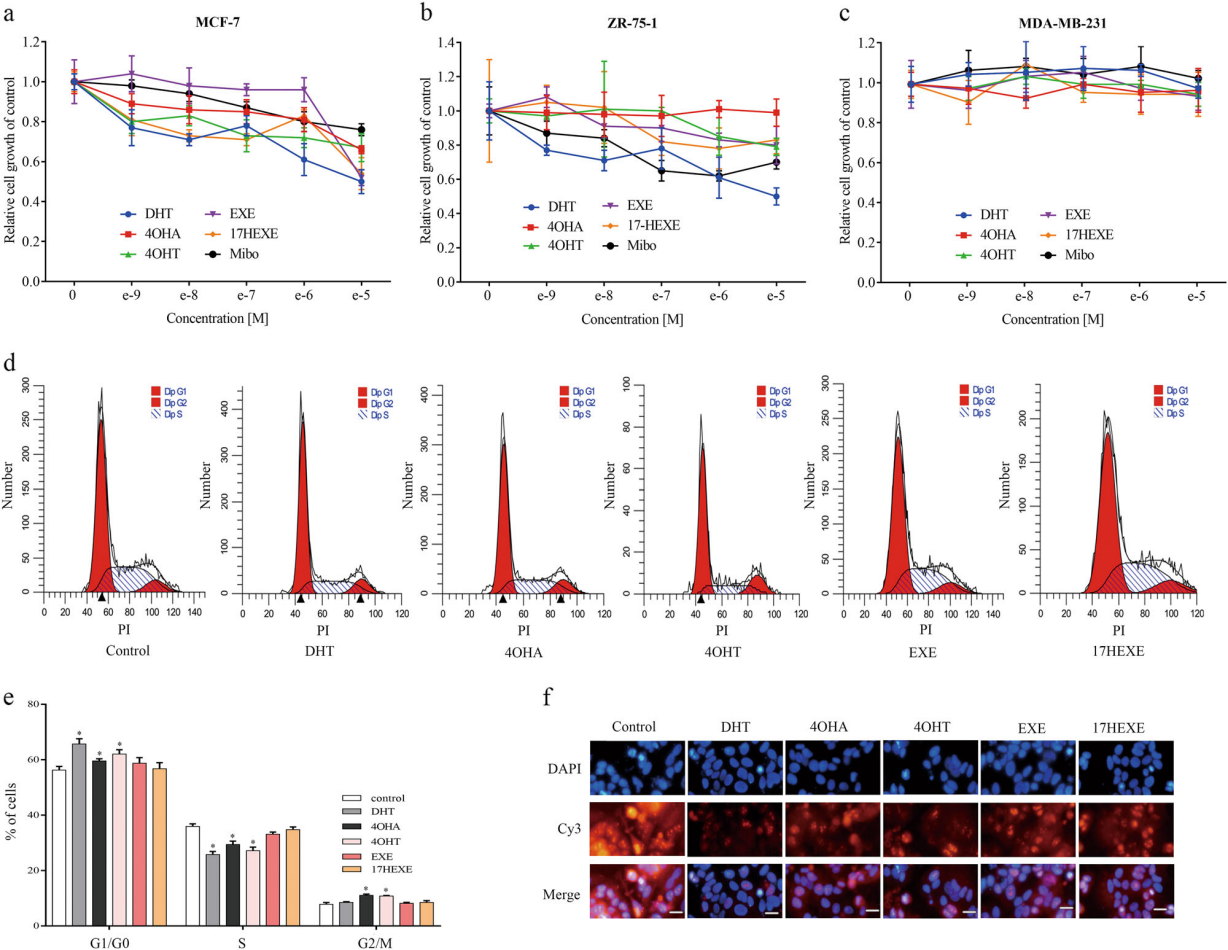
Proliferative response of cancer cells to indicated compounds. **a**–**c** the effect of concentrations of different compounds on the growth of the cells. Cells were cultured in 10% PRF-CT with E_2_ 1 nM (MCF-7 and ZR-75-1) or without E_2_ (MDA-MB-231) for 3 days before plating. Cells were seeded in 96-well plates and 24 h later they were exposed to different concentrations (1 nM– 10 μM) of compounds for 6 days (MCF-7 and ZR-75-1) or 3 days (MDA-MB-231). Cell growth was measured as described in the “Materials and methods”. The proliferation of cells is expressed as the ratio of the cells compared with the control wells (untreated cells). **d**, **e** Influence of different compounds on the cell cycle of MCF-7 cells. MCF-7 cells were cultured in 10% PRF-CT with E_2_ (1 nM) for 3 days before plating. Cells were seeded in six-well plates and, the next day, starved in PRF for 24 h. Cells were exposed to different compounds at a concentration of 100 nM for 72 h, and then subjected to cell-cycle analysis as described in “Materials and methods”. **f** MCF-7 cells on poly-l-lysine-coated glass coverslips were exposed to compounds (100 nM) in 10% PRF-CT with 1 nM E_2_ for 72 h. Control cells were treated with vehicle. DAPI stains DNA, and the secondary anti-mouse antibody to detect the antibody bound to Ki-67 is labeled with the cyanine dye Cy3. The cells were stained with DAPI (blue). Immunofluorescence staining of Ki-67 of cells was observed using fluorescence microscope. Red: Ki-67; blue: nucleus.Typical image is shown. Scale bars: 10 μm. Data represent a mean ± SEM of three independent experiments, each in triplicate; bars, SEM. *P ≤ 0.05 vs. control

To investigate the role of tested compounds on the proliferation of breast cancer cells, the growth of ER(+)/AR(+) breast cancer cells was measured after several days of drug treatment in 10% PRF–CT DMEM with 1 nM E_2_. As expected, the strong androgen DHT inhibits the proliferation of MCF-7 and ZR-75-1 cells at all tested concentrations when compared with controls (untreated cells; Fig. 2a, b). In addition, the inhibitory effect of DHT on the two cell lines is dose-dependent. Mibolerone is antiproliferative in two ER(+)/AR(+) cell lines at all concentrations. The same holds true for 17-HEXE. Exemestane is less effective. 4-OHT is strongly antiproliferative in MCF-7 cells and it inhibits the proliferation of ZR-75-1 cells only at micromolar concentrations, whereas the parental substance 4-OHA shows some antiproliferative action in MCF-7 cells and has no effect on the growth of ZR-75-1 cells (Fig. 2a, b). All the drugs have no effect on ER(−)/AR(−) MDA-MB-231 cells (Fig. 2c).

The antiproliferative action of tested compounds correlates well with the results of cell-cycle analysis in MCF-7 cells. MCF-7 cells are used as a model to study the androgenic property of compounds in subsequent experiments, because all the tested compounds decrease the growth of MCF-7 cells. As shown in Fig. 2d, e, the strong androgen DHT has the most significant influence on the cell cycle, increasing the proportion of MCF-7 cells in the G1/G0 phase and decreasing the proportion in the S-phase. With regard to this, most effective of the tested compunds is 4-OHT. Even 4-OHA is apparently more effective than Exemestane and 17-HEXE. The analysis of Ki67 expression further demonstrates the antiproliferative action of tested compounds in MCF-7 cells. In contrast to untreated cells, Ki67 expression is significantly reduced by all tested compounds (Fig. 2f).

### Modulation of AR protein levels by tested compounds

**Fig. 3:**
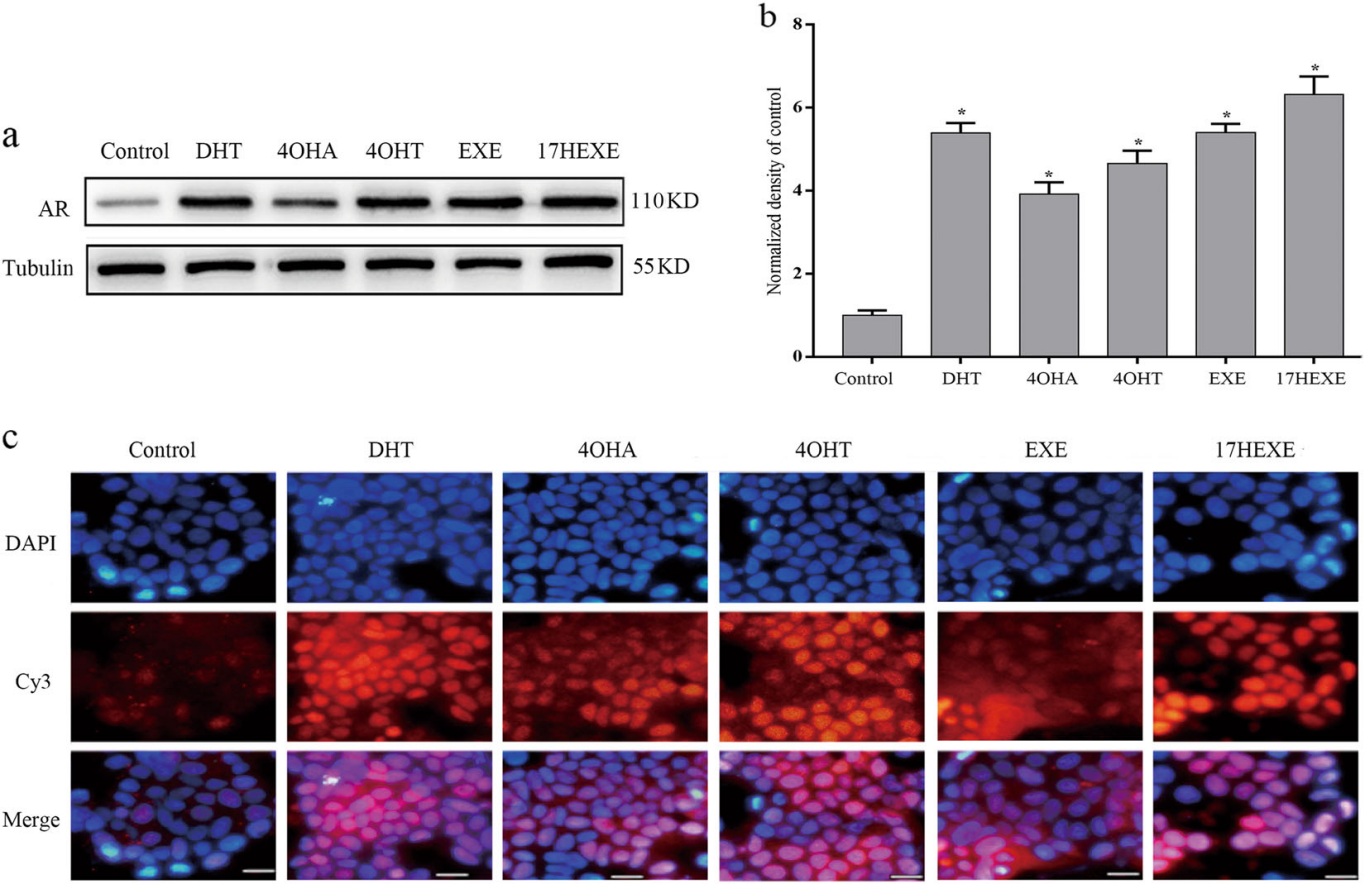
Influence of indicated compounds on the expression of the AR. All tested compounds increase the expression of the AR as demonstrated by western-blot and its densitometric evaluation and immunofluorescence in MCF-7 cells. **a** Cells culture method and treatment were same as shown in Fig. 2a. AR protein expression was analyzed by western blot as described in “Materials and methods”. **b** The densitometric evaluation of the bands is calculated by ImageJ. The numbers represent the densitometric value relative to untreated control. These data is representative of three separate experiments, each in triplicate; bars, SEM. *P ≤ 0.05 vs. control. **c** Immunofluorescence detected the AR protein expression as Ki67 shown in Fig. 2e. Typical image is shown. Scale bars: 10 μm

The binding of androgens to the AR increases the stability of the protein, transiently elevating AR protein levels in a variety of cell types^22^. The expression of the AR in MCF-7 cells usually is rather modest as can be seen in Fig. 3a, b. It can be enhanced considerably by incubation with DHT for 3 days. Surprisingly Exemestane is considerably more effective than Formestane. The western blot results are complemented by immunofluorescence results showing a drastic increase of the AR expression in MCF-7 cells caused by the incubation with the AIs and their derivatives. Immunofluorescence results also show a translocation of liganded AR into the nucleus. In contrast to the control, most AR proteins locate in treated cells nucleus (Fig. 3c). Apparently the induction of the AR is an additional feature of these substances which are able to inhibit the proliferation of ER(+)/AR(+) breast cancer cells through activating AR as androgens.

### Binding of the tested compounds to the AR and their regulation of AR transcriptional activity

**Fig. 4:**
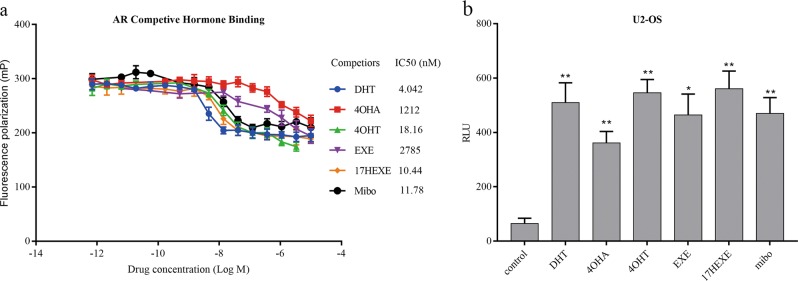
The affinity of indicated compounds to the AR and their regulation of AR transcriptional activity. **a** AR fluorescence polarization-based competitive hormone-binding assays. Rat AR ligand-binding domains tagged with a His glutathione S-transferase epitope (His-GST-ARLBD) were used at final concentrations of 25 nM. The fluorescently labeled AR ligand, Fluormone AL Red, was used at a final concentration of 2 nM. The competing test compounds were DHT, 4-OHA, 4-OHT, 17-HEXE, EXE, and Mibo as indicated. Point, mean of triplicate determinations; bars, 95% confidence intervals. Curve fitting was done using GraphPad Prism software (version 7.00). IC_50_s corresponding to a half-maximal shift in polarization values of the test compounds were determined using the maximum and minimum polarization values of the DHT-competitive-binding curve for AR. **b** Tested compounds regulate AR transcriptional activity at a concentration of 100 nM. ARE(3×)-regulated dual-luciferase activity in U2-OS cells under steroid-free conditions. Cells were transiently transfected with pcDNA3.1-AR and ARE3-luc2P (firefly luciferase reporter plasmids) and the internal normalization control pRL-TK (Renilla luciferase reporter plasmid). Six hours after transfection, cells were treated as indicated. Then they were assayed 48 h after transfection for dual-luciferase activity. Data shown are the mean of triplicate determinations and associated SEM; bars, SEM. *P ≤ 0.05, **P ≤ 0.01 vs. control

To confirm that steroidal AIs and their 17-OH derivatives reduce the growth of ER(+) breast cancer cells by activating AR, we investigate whether these compounds can bind to AR protein and promote AR transcriptional activity. As shown in Fig. 4a, 17-OH derivatives of Exemestane and Formestane competitively bind AR with an IC50 of 10.44 and 18.16 nM, respectively. Their affinity to the AR is almost as high as that of DHT (IC50, 4.02 nM). In contast, the affinity of the drugs Exemestane and Formestane to the AR is <1% that of DHT. It is therefore conceivable that the antiproliferative functions only arise after the sterols have undergone some intracellular metabolism. It is evident from the results of the reporter gene assays using U2-OS cells that binding to the AR also means activiation of the AR (Fig. 4b). In U2-OS cells not only the substances binding with high affinity to the AR can activate it, but Exemestane and Formestane also cause a significant activation of the AR. U2-OS cells express enzymes participations in the metabolism of sterols. Sonneveld reported that 17β-HSD type 5 (with 17β-ketosteroid reductase activity) is expressed in U2-OS cells^23^.

### The increase in wet weights of tested compounds

Development and growth of seminal vesicles (SVs) and prostate (P) depend on the presence of testosterone secreted by the testicles. Castration of adolescent male rats causes an shrinkage of these organs. The same holds true for the levator ani (LA) muscle. The weights of these organs can be reconstituted by constant adminstration of androgenic substances to the animal. The required dose to reconstitute P and SVs is a measure of the androgenic potency of the substance, and its anabolic influence on the levator muscle is called myotrophic effect.

**Fig. 5:**
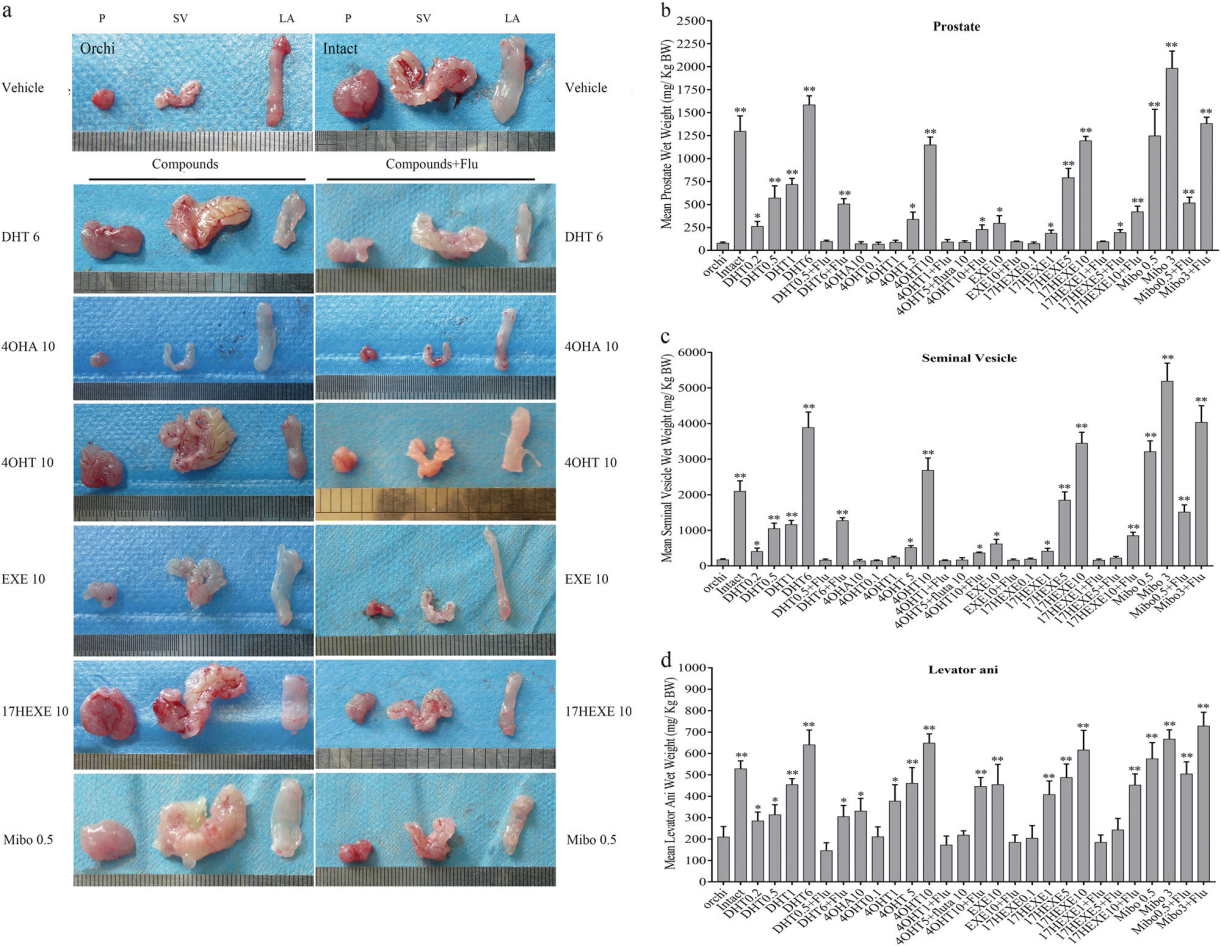
Estimation of the androgenic and myotrophic activities of the indicated compounds obtained by the Hershberger Assay. Wistar male rats were orchiectomized (orchi) or sham-operated (intact) under ether anesthesia. After 14 days of endogenous hormonal decline, animals were randomly allocated to treatment and vehicle groups (*n* = 6). The numbers at the end of the abbreviations correspond to the dose in mg/kg body weight of the animal. Flutamide was given by gavage (10 mg/kg/day). DHT dihydrotestosterone, Flu flutamide, 4OHA 4-hydroxyandrostenedione, 4OHT 4-hydroxytestosterone, EXE Exemestane, 17HEXE 17-hydroexemestane, Mibo Mibolerone. Then the animals were treated once a day. The rats were sacrificed after completion of the 12-day treatment. Following removal, wet weights of the prostate (P), seminal vesicle (SV), levator ani muscle (LA) were determined. **a** Size and shape of P, SV and LA at the end of the treatment period. **b**, **c**, and **d**, influence of treatment with the compounds alone or together with the antiandrogen flutamide at the indicated doses on the weight of the organs (P, SV and LA) of the castrated animals. The mean wet weight is represented by the bars in the graphs. Values are means ± SEM; bars, SEM. *P ≤ 0.05, **P ≤ 0.01 vs. control

Figure 5a shows the sizes of P, SVs, and LA of intact male rats and of castrated rats obtained after 12 days of no treatment (contols (orchi)) or after subcutaneous injection of different doses of substances with presumed androgenic and/or myotrophic potencies.

It is evident that castration affects the size of all three organs strongly. DHT in a dose of 6 mg/kg can reconstitute the organs completely. This can be prevented by the antiandrogen Flutamide. The synthetic androgen Mibolerone (0.5 mg/kg) is more effective than DHT. 4-OHA at a dose of 10 mg/kg has no effect on P and SVs. At the same dose Exemestane is slightly androgenic. The 17-hydroxy derivatives of 4-OHA and Exemestane are strong androgens. All androgenic effects can be eliminated by concomitant oral administration of Flutamide.

The results of the visual inspection were quantified by recording the wet weight of the organs as a result of the treatment conditions (Fig. 5b–d). It can be seen that 17-HEXE is a little more androgenic than 4-OHT and that 4-OHT is a little more myotrophic than 17-HEXE. The results of the Hershberger Assay confirm the androgenic potential of Formestane and Exemestane provided they are modified to their 17-hydroxy derivatives.

### The role of AR on the proliferation of breast cancer cells

**Fig. 6:**
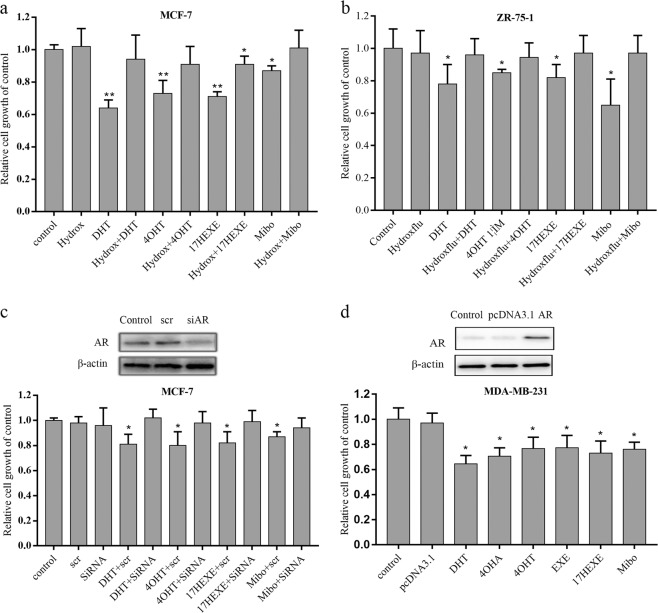
Effects of blocking of the AR or regulation of its cellular expression on the ability of compounds to influence the proliferation of breast cancer cells. Cells were cultured and treated as described in the legend of Fig. 2. Cell growth was measured as described in “Materials and methods”. **a** and **b** Opposing effect of hydroxyflutamide (100 nM) given simultaneously to the inhibitory effect of test compounds (100 nM) on the growth of MCF-7 and ZR-75-1 cells. **c** and **d** The influence of changes in expression of the AR in MCF-7 cells (downregulation) and MDA MB-231 cells (upregulation) on the proliferative response to the tested compounds (100 nM). In MCF-7 cells inhibition of proliferation is mitigated, but in MDA-MB-231 cells proliferation is reduced. The blot shows the expression of the AR protein. Cell growth is expressed as the ratio of the number of cells in the experimental well to that in control wells (untreated cells). The columns represent the mean of three experiments, each with five replicates; bars, SEM. *P ≤ 0.05, **P ≤ 0.01 vs. control

The antiproliferative effect of DHT on MCF-7 and ZR-75-1 cells is clearly mediated by the AR. Correspondingly it can be abolished by substances inhibiting the binding of DHT to the AR. One such substance is OH-Flutamide, the active metabolite of Flutamide. When 100 nM DHT and 100 nM OH-Flutamide together are incubated with MCF-7 or ZR-75-1 cells, the antiproliferative effect of DHT is almost completely abolished (Fig. 6a, b). 4-OHT and 17-HEXE at 100 nM concentration behave like DHT. Their antiproliferative effect on MCF-7 and ZR-75-1 cells is clearly androgenic.

We also used siRNA to downregulate AR gene expression in MCF-7 cells. AR downregulation diminishes the ability of 100 nM DHT to inhibit the proliferation of MCF-7-cells. More impotrantly, it gives identical results with 4-OHT and 17-HEXE, thereby proving that the antiproliferative effects of the two metabolites of steroidal AIs are due to AR activation (Fig. 6c). The proliferation of MDA-MB-231 cells is completely insensitive to all the tested compounds (Fig. 2c). After transfection with the AR these cells become sensitive to inhibitory effects of these substances (100 nM) (Fig. 6d). This is an additional proof that all these substances activate the AR. The antiproliferative effect of the AR can be induced by the introduction of AR in AR(−) breast cancer cells.

### The decrease of cyclin D1 expression by Activated AR

**Fig. 7:**
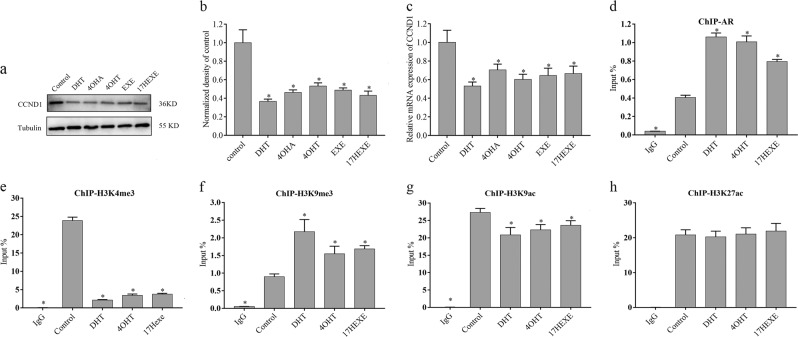
The effect of activated AR on *CCND1* expression by changing histone modifications of its promoter. Cell culture method and treatment was as shown in Fig. 2d. The experimental protocol was as described in “Materials and methods”. **a** and **b** The expression of the cyclin D1 protein. Blots were also probed for tubulin (bottom) to verify equal amounts of protein loaded in each lane. The densitometric evaluation is calculated by ImageJ. **c** The abundance of cyclin D1 mRNA has been detected by real-time reverse transcription-PCR, as described in “Materials and methods”. **d**–**h** MCF-7 cells were grown in 10 cm dishes. Confluent cultures (80%) were shifted to PRF for 24 h and then treated with indicated compounds at a concentration of 10^−7^ M or left untreated in PRF-CT for 2 h. ChIPs were carried out on serum starved MCF-7 cells as described in “Materials and methods”. Cells were lysed and proteins were precipitated using antibodies against AR (**d**), H3K4me3 (**e**), H3K9me3 (**f**), H3K9ac (**g**), H3K27ac (**h**) or rabbit IgG (2 μg/sample each). In control samples (Ig), normal rabbit IgG was used instead of the primary antibodies as control of antibody specificity. Inputs DNA were amplified as loading controls. Five regions of *CCND1* promoter were examined by quantitative PCR analysis. The distance from TSS of the *CCND1* promoter portions is indicated in Table 1. The region containing the ARE site was detected by PCR with specific primers (p1-ARE) for antibodies of AR and H3K4me3. Specific primers P4 and p5 were used to investigate the changes in methylation or acetylation of H3K9. H3K27 acetylation does not change as judged by using all pairs of primers. These data are representative of three separate experiments, each in triplicate; bars, SEM. *P ≤ 0.05 vs. control

DHT decreases the proliferation of MCF-7 cells. This is accompanied by a reduction of the expression of cyclin D1^20^. We wanted to find out whether the inhibition of the growth of ER(+)/AR(+) breast cancer cells of the other investigated sterols also results from down-regulation of cyclin D1. For this purpose, serum-starved MCF-7 cells were left untreated or treated with 100 nM tested compounds for 72 h, and cyclin D1 expression was assessed by real-time RT-PCR and western blot analysis. As shown in Fig. 7, DHT causes a decrease in Cyclin D1 mRNA (Fig. 7c) and protein (Fig. 7a, b) if compared with cells grown without test compounds. The androgenic metabolites and even the mother substances have the same effect.

It is reported that DHT liganded AR interacts with an ARE in the promoter of CCND1 gene and causes a decrease of CCND1-promoter activity in MCF-7 cells and ultimately reduces the gene expression^20^. DHT stimulation induces the recruitment of HDAC1 to the CCND1-ARE consensus sequence. It results in a deacetylation of histone by DHT liganded AR. To verify whether 4-OHT and 17-HEXE mediate CCND1 gene expression by epigenetic regulation similar to DHT, we investgated the enrichment of AR, H3K4me3, H3K9me3, H3K9ac, H3K27ac binding to the *CCND1*-promoter. As shown in Fig. 7d, treatment of MCF-7 cells with DHT causes a strong increase of AR binding to ARE site of *CCND1*-promoter, as demonstrated by ChIP-qPCR. The effect of the 4-OHT and 17-HEXE is almost as strong as that of DHT (Fig. 7d). MCF-7 cells were incubated with 4-OHT or 17-HEXE for two hours. Thereafter chromatin immunoprecipitation (ChIP) of methylated or acetylated histones was performed. Analysis of the coprecipitated DNA fragments shows clearly that H3K4me3, which indicates transcription of a gene, in this case *CCND1*, is <20% of the untreated control cells (Fig. 7e). H3K9me3, a marker of gene repression, is increased (Fig. 7f). They are decreased (H3K9ac) or unchanged (H3K27ac) (Fig. 7g, h). These results are a clear indication that not only DHT but also the androgens derived from Formestane and Exemestane cause a down-regulation of cyclin D1.

### The effects of sterols on the DMBA-induced mammary carcinoma in rats

**Fig. 8:**
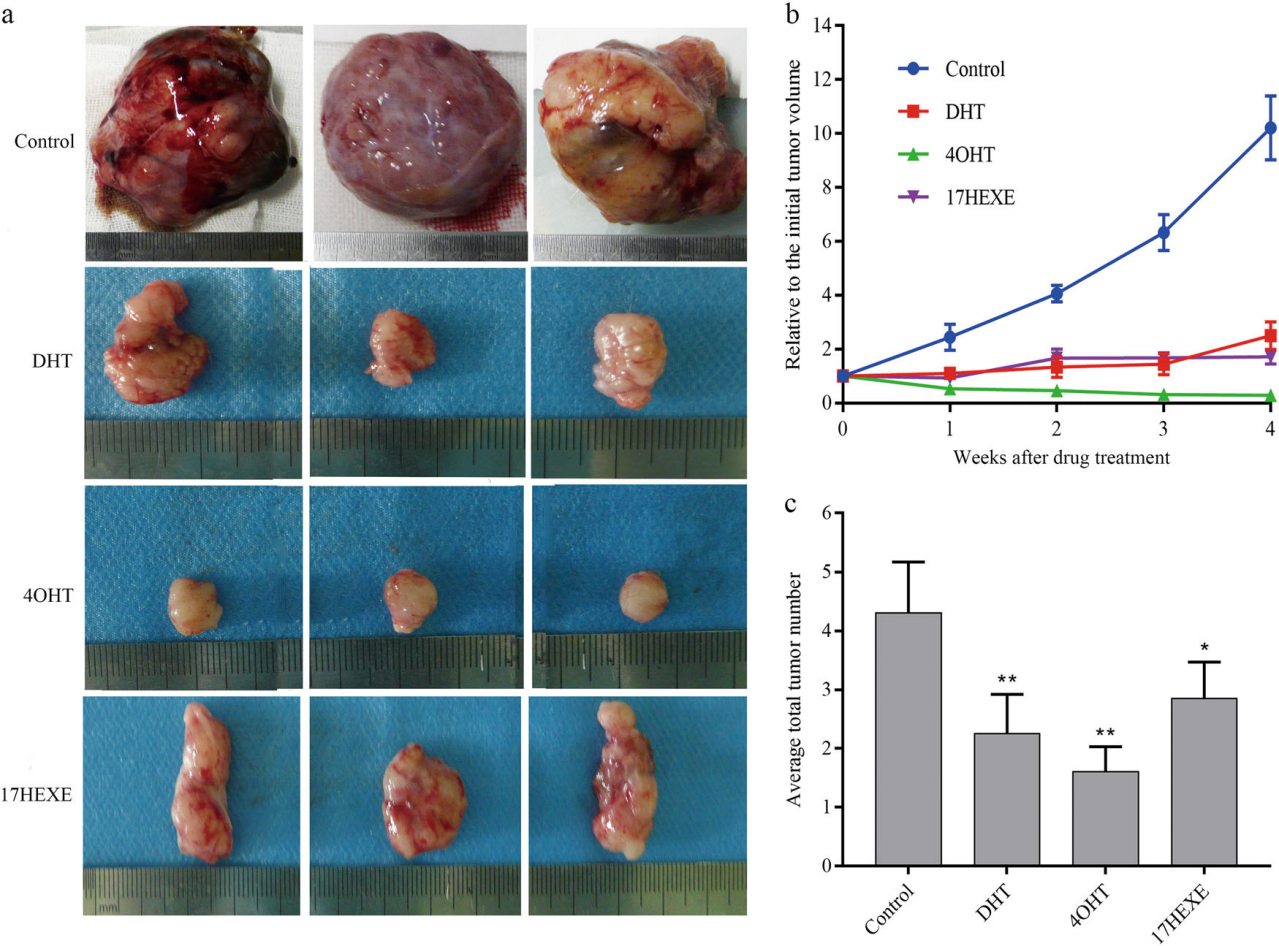
The effects of sterols on the DMBA-induced mammary carcinoma in rats. At an age of 50–54 days, female Sprague-Dawley rats were dosed intragastrically with 20 mg DMBA. when at least one tumor measuring 1 cm in diameter was found, the rats were ovariectomized and given intramuscular 1 μg/rat E_2_ daily. Animals were selected for experiments when at least one tumor per rat had reached a diameter of 1.5 cm. The skin covering the tumor was shaved and animals were divided into four groups. The tumors were treated by direct application of DHT, 4-OHT, and 17-HEXE. Untreated tumors served as controls. **a** Representative images of the tumors. **b** Tumor growth curve. **c** Average number of tumor nodules before and after treatment.The data represent six sets of independent experiments and are shown as the means ± SD. *P < 0.05, **P ≤ 0.01 vs. control group

To confirm that steroidal AIs and their 17-OH derivatives also inhibit the growth of breast cancer cells in vivo, we produced breast cancers in female rats by oral application of DMBA. Cancer-bearing rats were ovariectomized and received hormone replacement therapy with E_2_. The cancers were treated topically with DHT, 4-OHT, and 17-HEXE. Figure 8 shows an increase in size of the tumors in control group A, whereas in the treatment groups B and C, DHT and 17-HEXE cause a growth arrest and even 4-OHT causes a shrinkage of the tumor (Fig. 8a, b).

The average tumor number is, for all groups, 1.2 ± 0.15 tumors per rat at the beginning of the treatment. Values of 4.3 ± 1.2, 2.25 ± 0.8, 1.6 ± 0.4, and 2.85 ± 0.68 were found after 28 days of treatment with control, DHT, 4-OHT, and 17-HEXE treated animals, respectively (Fig. 8c). All drugs significantly decrease the number of new tumors at the end of the treatment period compared to control (*P* < 0.01 for DHT and 4-OHT, *P* < 0.05 for 17-HEXE). Of these drugs, animals in 4-OHT group almost did not develop new tumors after treatment.

## Discussion

There is a lack of cross-resistance between aromatase inhibitors and inactivators. This lack has been termed “puzzling”^24^. We think that it is likely that the interaction of metabolites with the AR can explain why these substances still work, when the nonsteroidal AIs have failed. The results of reporter gene assays show that these metabolites can activate the AR. It is well known that human 3a-hydroxysteroid dehydrogenases play central roles in steroid hormone metabolism and action. Only AKR1C3 with 17β-ketosteroid reductase activity reduced △4-androstene-3,17-dione to testosterone. AKR1C3 is most prominent in mammary glands and MCF-7 cells^25,26^. Therefore, in MCF-7 cells Formestane and Exemestane are converted into one or more different metabolites that can activate the AR.

Androgens have been successfully used to treat advanced breast cancer. These early results gave rise to the idea that the growth of breast cancer cells can be inhibited by androgens. Correspondingly we tested the ability of Formestane and Exemestane, as well as that of their androgenic metabolites to inhibit the proliferation of MCF-7 or ZR-75-1 cell lines. It is known that the androgen DHT inhibits the proliferation of E_2_-stimulated MCF-7 and ZR-75-1 cell lines^17–19^. To our surprise not only the androgens 4-OHT and 17-HEXE but also the original drugs were able to inhibit the growth of these cells. Since both drugs do not bind avidly to the AR^15,27,28^ it is obvious that they gave rise to 4-hydroxy-C-19 steroids able to interact with the AR. Since the growth-inhibiting effect of DHT, 17-HEXE, and 4-OHT could be abolished by hydroxy-flutamide and AR siRNAs, we can safely state that the antiproliferative effects are mediated by the AR. This also holds true to metabolites originating from Formestane and Exemestane made in the cancer cells. This mechanism may explain the lack of cross-resistance between steroidal and nonsteroidal AIs.

In addition to the androgenic inhibitory effects, the drugs and their metabolites induce the expression of the AR in MCF-7 cells. This theoretically is a reinforcing effect possibly facilitating apoptosis. DHT has apoptotic effects on ZR-75-1 cells. The extent of these is concentration-dependent^29^.

Since both drugs clearly give rise to androgens when incubated within the appropriate cells, we wanted to investigate whether this is also the case in intact animals. The classical method to test whether some substances can exert androgenic effects is the Hershberger assay. Castration of immature male rats leads to a shrinkage of the main androgen-dependent organs like P and SVs and also of an androgen-dependent muscle, LA. Administration of androgens will reconstitute P and SVs. Corresponding animal experiments have been undertaken also with Formestane^30^ and Exemestane^28^ but not with their 17-OH derivatives. In our hands both drugs hardly show any androgenic effect. This agrees with the literature where in case of Formestane 8 mg/rat (corresponds roughly to 24 mg/kg) showed 1% of the androgenic activity of testosterone, in case of Exemestane a slight androgenic activity was confirmed at doses of 3 and 10 mg/kg^30^. We found the same effect for Exemestane (10 mg/kg) and no effect on androgenic organs whatsoever for subcutaneous doses of 10 mg/kg of Formestane. Both drugs are showing obvious myotrophic effects. The almost complete lack of androgenic effects in the castrated rat may be due to the rapid phase II metabolism of the substances. Whereas Formestane can be directly conjugated with glucuronic acid in the liver and escape reduction of the C17-keto-group, Exemestane is rendered a little androgenic before it can be conjugated with glucuronic acid.

Next we wanted to have a closer look at the mechanism responsible for the growth retardation of the breast cancer cells. Analysis of the cell cycle by flow-cytometry shows clearly an arrest of MCF-7 cells in the G1/G0 phase and concomitant slight depletion of cells in the S-phase, if the cells are treated with the drugs or their androgenic metabolites. This retardation correlates well with a decreased expression of the proliferation-marker Ki67.

The tested androgens clearly have an effect on the cell cycle. Apparently androgens are offering a way to reduce the expression of cyclin D1 in breast cancer cells thereby retarding the cell cycle. We can demonstrate that the steroidal AIs and their androgenic metabolites as well as DHT cause a down-regulation of cyclin D1 expression. A possible mechanism is located in changes in the structure of chromatin caused by androgens, which impedes the transcription of *CCND1*.

Recently, we were able to demonstrate that the direct influence of Formestane on an ER(+) breast cancer in situ induces a regression of the tumor by more than 50% within 40 days. Besides inactivation of aromatase which evokes a reaction of the tumor rather slowly, different mechanisms must be responsible for this drastic and rather quick success. One important mechanism may be the influence on the cell cycle. Other mechanisms must be related to apoptosis. This will be the topic of future research.

Exemestane and Formestane have been proven useful in the treatment of DMBA-tumors. Their effect has been attributed to estrogen deprivation by inactivation of aromatase in the animal and in the tumor^30,31^. Lack of E_2_ is an impossible cause of tumor regression in our experiment, since the animals are receiving E_2_ and this is stimulating the growth of the tumors in the control animals considerably. The observed effects are independent from the prevailing E_2_-levels. The reaction to topical DHT shows that the effect may be androgenic, but the special success of topical 4-OHT most probably is due to a so far unidentified additional mechanism.

Our experiments have elucidated the antiproliferative effect of androgens on breast cancer cells. We could demonstrate that the steroidal aromatase inactivators formestane and exemestane are chemically modified within the cancer cells and that the modified sterols act predominantly as antiproliferative androgens. The lack of cross-resistance between steroidal and nonsteroidal aromatase inhibitors suggests that the drugs can be given in a sequential manner.

## Materials and methods

### Compounds and cell lines

4-OHT and 17-HEXE were provided by Chiracon GmbH 14943 Luckenwalde, Germany. All other compounds were obtained from Sigma-Aldrich. A total of four cancer cell lines were used in the present study: MCF-7, MDA-MB-231, ZR-75-1, and U2-OS. All cells were obtained from the cell bank of Chinese Academy of Sciences (Shanghai, China) after authentication by short tandem repeat profiling, and maintained as recommended. Cell culture reagents were from Invitrogen. All tested compounds were dissolved in dimethyl sulfoxide and added to the medium achieving a dilution of less than 1:1000 (v/v). Before each experiment, cells were grown in phenol red-free (PRF) medium containing 10% charcoal-treated fetal calf serum (PRF–CT) for 3 days and then in serum-starved PRF for 24 h to synchronize the cells. All the experiments were performed in 10% PRF–CT.

### Cell proliferation assays

Cells were seeded on 96-well plates (2 × 10^3^ cells/well) in 10% PRF–CT with E_2_ 1 nM (MCF-7 and ZR-75-1) or without E_2_ (MDA-MB-231). After 24 h, cells were exposed for 3–6 days to various concentrations of compounds (1 nM– 10 μM) or left untreated. The incubation time and the compounds concentrations were chosen based on previous studies^17,32–34^. The effects of the various drugs on cell proliferation at the indicated concentrations (1 nM– 10 μM) were determined by using a CCK8 kit (Dojindo Laboratories, Kumamoto, Japan) according to the manufacturer’s protocol. The absorbance at 450 nm was measured by using a Versamax microplate reader (Thermo Varioskan Flash, USA). Cell growth is expressed as the ratio of the cells compared with the control wells (untreated cells).

### Cell cycle analysis

Cell cycle kinetics were studied by flow cytometry. MCF-7 cells were cultured in 10% PRF–CT with 1 nM E_2_ for 3 days prior to seeding on six-well plates (10^5^ cells/well). Following seeding of cells, cells were serum starved for 24 h, and then treated with media containing 100 nM of the test compounds for 72 h. Cell-cycle analysis was performed as described by Ando^20^.

### Western blotting analysis

Total cell proteins were obtained from 70% confluent cell cultures. Protein extracts of whole cells (50 μg) were denatured, separated by SDS–PAGE and transferred to polyvinylidene fluoride membranes. After blocking with 3% bovine serum albumin in Tris-buffered saline-Tween, membranes were probed overnight at 4 °C. The following monoclonal (m) and polyclonal (p) antibodies (Ab) were used: anti-AR mAb (441, Santa Cruz Biotechnology, USA), anti-CCND1 mAb (92G2, Cell Signaling Technology, USA), α-Tubulin pAb (Beyotime Biotechnology, China), β-Actin Mouse mAb (4D3, Beyotime Biotechnology, China) and normal mouse or rabbit immunoglobulin G (Ig) (Santa Cruz Biotechnology, USA). After incubation with the appropriate secondary antibody, results were detected using ECL detection reagents. Immunoreactive bands were quantified by densitometry using ImageJ software.

### Immunofluorescence

MCF-7 cells were seeded on 24-well plates (2 × 10^4^ cells/well) with a poly-l-lysine-coated glass coverslip per well in 10% PRF–CT. After 24 h, cells were treated in a concentration of 100 nM compounds or left untreated for 72 h. Immunofluorescence was performed as described in literature^35^. In brief, cells fixed with 4% paraformaldehyde were permeabilized and blocked with 0.2% Triton X-100 and 5% FBS in PBS. Samples were then incubated with an antibody against AR (Santa Cruz) or Ki67 (Santa Cruz) in phosphate-buffered saline containing 0.1% triton overnight. Incubation with the secondary CY3-labeled anti-mouse antibody was performed in 2.5% bovine serum albumin for 2 h at ambient temperature. The nuclei were stained with 4′,6-diamidino-2-phenylindole (DAPI) (1 μg/ml) for 30 min. Cells were visualized with a ×40 objective and a Qimaging digital camera coupled to an Olympus X71 fluorescence microscope using a red fluorescent protein filter.

### Transient transfection with small interfering RNA

To confirm the target of the antiproliferative activity of compounds we downregulated and upregulated the expression of AR in MCF-7 and MDA-MB-231 cell lines, respectively. AR siRNA and its control siRNA used in this study were described by Wu et al. ^36^. The sequence of the AR siRNA used was 5′-CAAGAUCCUUUCUGGGAAATT-3′ and a nonspecific siRNA duplex was used as a negative control. Growing cells were switched to PRF for 24 h and then switched to 10% PRF–CT medium containing 1 nM E_2_ for 48 h. MCF-7 cells were trypsinized and transfected in suspension with 2.5 μg siRNA (siAR or scrambled siRNA) or pcDNA3.1-AR in six-well plates, using Lipofectamine 3000 (Invitrogen) in OPTI-MEM medium (Gibco), following the manufacturer’s instructions. Cells were incubated with the siRNA or pcDNA3.1-AR Lipofectamine3000 complex at 37 ℃ for 6 h and then switched to fresh 10% PRF-CT with or without tested compounds (10^–7^ M) for 72 h before analysis.

### Competitive receptor-binding assays

Competitor assay kit (PolarScreen™ AR Competitor Assay, Red; Invitrogen) was used according to manufactuer’s protocol to measure competitive ligand binding to AR. Briefly, compounds were dissolved in DMSO and mixed at 2% (vol/vol) with AR Red Screening Buffer. The tested compounds were subsequently serially diluted in two-fold increments with AR Red Screening Buffer and mixed 1:1 (vol/vol) with a 2 × AR/Fluormone AL complex. Competition for binding AR between Fluormone AL and the tested compounds was allowed to come to equilibrium for 4 h and not more than 20 h at room temperature in the dark. Fluorescence polarization value (mP) was measured on a fluorescence polarization plate reader (Biotech cytation5). Each compound dose was performed in triplicate, relative error was calculated from the standard error of the mean (SEM), and curve fitting was performed using GraphPad Prism™ 7.0 software.

### Reporter gene assays

The pGL4.27 and pRL-TK Renilla constructs were kindly provided by Dr. Haisu Wan (Medical laboratory center, Affiliated Hospital of Southwest Medical University, China). The reporter construct ARE3-luc2P, containing three tandem copies of a synthetic ARE, was constructed by pGL4.27. We also made an encoding full length AR vector, pcDNA3.1-AR. U2-OS cells were seeded into a 24-well plate (2 × 10^4^ cells/well) in PRF DMEM plus 10% charcoal-stripped fetal bovine serum. The following day, medium was removed and replaced with fresh 10% PRF–CT. Cells were transiently transfected in triplicate using Lipofectamine 3000 according to the manufacturer’s protocol with 60 ng pcDNA3.1-AR, 180 ng ARE3-luc2P, 12 ng pRL-TK (normalization vector). After 6 h of transfection, the medium was replaced with fresh 10% PRF–CT and incubated overnight and then treated with various ligands, as indicated in the legend of Fig. 4. Following 48 h, firefly luciferase activity was measured using dual luciferase assay system (Promega) according to the manufacturer’s manual, normalized to renilla luciferase activity and expressed as relative luciferase units.

### Chromatin immunoprecipitation

MCF-7 cells were grown in 10 cm dishes. Confluent cultures (80%) were shifted to PRF for 24 h and then treated with various drugs at a concentration of 10^–7^ M (as shown in Fig. 8) or left untreated in 5% PRF-CT for 2 h. Following treatment, ChIP assay was performed as described by Morelli et al. ^37^ and using a simple Chip plus Sonication Chromatin IP kit (Cell Signaling Technology, #56383), following the manufacturer’s protocol. The cells were sonicated 10 times for 10 s at 20% of maximal power (Fisher Sonic Dismembrator) and then treated according to the protocol. Anti-AR (Cat. No. 5153), anti-H3K4me3 (Cat. No. 9751), anti-H3K9me3 (Cat. No. 13969), anti-H3K9ace (Cat. No. 9649), anti-H3K27ace (Cat. No. 8173) or rabbit IgG (Cat. No. 3900) of Cell Signaling Technology antibody were used to precipitate chromatin fragments from cell extracts. We used real-time quantitative PCR (SYBR green) to amplify the DNA fragment in the antibody precipitated DNA and the unprecipitated input DNA to calculate ΔCT values. A specific primer pair was used to amplify 134 bp of the ARE-containing cyclin D1 promoter fragment. For the histone modification antibody, another four pairs of prime locating at 1000 to −2000 bp region from the transcription initiation site of CCND1 were used besides the prime containing ARE. All primers are listed in the Table 1. The *R*_Q_ values (*R*_Q_ = 2^–ΔCT^) are presented and reflect the precipitated DNA as a percentage of the input DNA.

**Table 1:** the primers of *CCND1* promoter for Chip-seq

Name	Orientation	Sequence(5′−3′)	The site of CCND1 promoter
P1-ARE	Forward	CGCCGGAATGAAACTTGC	−586 to −453 bp
	Reverse	ACAGACGGCCAAAGAATCTCA	
P2	Forward	GGCGATTTGCATTTCTATGA	−204 to +25 bp
	Reverse	CAAAACTCCCCTGTAGTCCGT	
P3	Forward	AGTGGGCGAGCCTCTTTAT	−1888 to −1734 bp
	Reverse	GCCTGGATGATTTATGGGG	
P4	Forward	CGAAGTGGAAACCATCCG	+234 to +368 bp
	Reverse	AGGACCTCCTTCTGCACACATT	
P5	Forward	TCGCATCTTGCTGTGAGCA	−1451 to −1348 bp
	Reverse	TTCAGTGTCATCAAACCACCGT	

### RNA isolation and real-time PCR

MCF-7 cells were cultured under 10% PRF–CT with 1 nM E_2_ for 3 days prior to seeding on six-well plates (10^5^ cells/well). Following seeding of cells, cells were serum starved for 24 h, and then treated with media containing the test compounds 100 nM for 3 days. Total RNA was isolated using TRIzol reagent (Invitrogen, USA) according to the manufacturer’s instructions and treated with DNase I (Thermo Scientific™, Cat. No. EN0521). RNA (1 μg) was reverse-transcribed using the PrimeScript™ RT reagent Kit (Takara, Code No. RR037A). RT-PCR was performed using the Applied Biosystems StepOne Real-Time PCR System and TB Green™ Fast qPCR Mix (Takara, Code No. RR430A). *CCND1* Primers used for the amplification were 5′-CGTGGCCTCTAAGATGAAGGA-3′ (forward) and 5′-CGGTGTAGATGCACAGCTTCTC-3′ (reverse). Negative controls contained water instead of first strand cDNA. The human GAPDH (forward, 5′-ATGCTGGCGCTGAGTACGTC-3′, reverse, 5′-GGTCATGAGTCCTTCCACGATA-3′) gene was used as an internal reference. The expression level was normalized to the GAPDH control, and relative expression values were determined against the untreated sample using the 2^–^^ΔCT^ method.

### In vivo experiments

The animal studies were conducted under the approval by the Experimental Animal Management Committee of the affiliated Hospital of Southwest Medical University, Luzhou, China. Animals were obtained from Chengdu Da Shuo Biotechnology co. Ltd (China) and were maintained under controlled conditions of temperature (20 ± 1 °C, relative humidity 50–80%) and illumination (12 h light, 12 h dark). All rats had free access to a standard rat diet and water.

### Hershberger assay

The Hershberger assay was performed according to the guidelines of the rodent Hershberger assay^38^. Male Wistar rats (140 g, 5–6 weeks) were orchiectomized (orchi) or sham-operated (intact) under ether anesthesia. After 14 days of endogenous hormonal decline, animals were randomly allocated to treatment and vehicle groups (*n* = 6); orchi group = orchi rats receiving sham injections; intact group = sham-operated rats receiving sham injections; treatment group = orchi rats receiving drugs injections by subcutaneous. The antiandrogen flutamide given by gavage (10 mg/kg/day). Drugs and doses are indicated in Fig. 5. Drugs were dissolved in ethanol and diluted in corn oil. The animals were treated once a day. Rats were sacrificed after completion of the 12-day treatment. Following removal, the wet weights of the P, SV, and LA muscle were determined. The data of tissue weights are presented as mean ± SEM.

### Mammary tumor study

At an age of 50–54 days, female Sprague-Dawley rats were dosed intragastrically with 20 mg DMBA (Sigma Chemical Co.) dissolved in peanut oil (1 ml/rat). Starting at 40 days after DMBA treatment, animals were examined weekly by palpation; when at least one tumor measuring 1 cm in diameter was found, the rats were ovariectomized using ether anesthesia and were given E_2_ (1 μg/rat) by daily intramuscular injection. Animals were selected for experiments when at least one tumor per rat had reached a diameter of 1.5 cm. Tumors were measured with calipers once a week, and their volume was calculated according to the following formula: volume = length × (width)^2^ × 0.5. Rats were then divided into four groups of six animals each. The tumors of group A received 1 ml of sterol-free control cream without anything twice a day via topically application; tumors in group B were treated with 1 ml of cream containing 2.5% DHT twice daily; tumors in group C received a cream containing 2.5% 4-OHT; group D was treated with 2.5% 17-HEXE. All creams were applied for 4 weeks. Animals that failed to develop tumors by day 150 were discarded. Tumor growth in the control and treated groups was expressed as a value relative to the initial tumor volume, measured on the 1st day of treatment and taken as 1. At the beginning and end of the treatment period, the number of tumor nodules was counted.

### Statistical analysis

All data obtained from at least three independent experiments were expressed as the mean ± SEM and analyzed using one-way analysis of variance (ANOVA), followed by the LSD post hoc test for multiple comparisons (SPSS 11.5 statistical software). *P* < 0.05 was considered significant.

## References

[1] Carey LA (2006). Race, breast cancer subtypes, and survival in the Carolina Breast Cancer Study. JAMA.

[2] Murase K (2014). Biological characteristics of luminal subtypes in pre-and postmenopausal estrogen receptor-positive and HER2-negative breast cancers. Breast Cancer.

[3] Ahmad, I. Tamoxifen a pioneering drug: an update on the therapeutic potential of tamoxifen derivatives. Eur. J. Med. Chem. **143**, 515–531 (2018). 10.1016/j.ejmech.2017.11.056 29207335

[4] Riemsma R (2010). Systematic review of aromatase inhibitors in the first-line treatment for hormone sensitive advanced or metastatic breast cancer. Breast Cancer Res. Treat..

[5] Dhillon S (2013). Everolimus in combination with exemestane: a review of its use in the treatment of patients with postmenopausal hormone receptor-positive, HER2-negative advanced breast cancer. Drugs.

[6] Coombes RC, Dowsett M, Goss P, Gazet J, Brodie A (1984). 4-Hydroxyandrostenedione in treatment of postmenopausal patients with advanced breast cancer. The Lancet.

[7] Labrie F (1991). Intracrinology. Mol. Cell. Endocrinol..

[8] Covey DF, Hood WF (1982). A new hypothesis based on suicide substrate inhibitor studies for the mechanism of action of aromatase. Cancer Res..

[9] Carlini P (2001). Formestane, a steroidal aromatase inhibitor after failure of non-steroidal aromatase inhibitors (anastrozole and letrozole): Is a clinical benefit still achievable?. Ann. Oncol..

[10] Carlini P (2003). Is there a benefit by the sequence anastrozole–formestane for postmenopausal metastatic breast cancer women?. J. Steroid Biochem. Mol. Biol..

[11] Carlini P (2007). Clinical evaluation of the use of exemestane as further hormonal therapy after nonsteroidal aromatase inhibitors in postmenopausal metastatic breast cancer patients. Cancer Invest..

[12] Stebbing J, Gaya A (2001). The evidence-based use of induction chemotherapy in breast cancer. Breast Cancer.

[13] Prat A (2015). Response and survival of breast cancer intrinsic subtypes following multi-agent neoadjuvant chemotherapy. BMC Med..

[14] Freedman O, Verma S, Clemons M (2005). Using aromatase inhibitors in the neoadjuvant setting: evolution or revolution?. Cancer Treat. Rev..

[15] Davies JH (1992). Effects of 4-hydroxyandrost-4-ene-3, 17-dione and its metabolites on 5α-reductase activity and the androgen receptor. J. Enzyme Inhib..

[16] Ariazi EA (2007). Exemestane’s 17-hydroxylated metabolite exerts biological effects as an androgen. Mol. Cancer Ther..

[17] Birrell S (1995). Androgens induce divergent proliferative responses in human breast cancer cell lines. J. Steroid Biochem. Mol. Biol..

[18] Poulin R, Baker D, Labrie F (1988). Androgens inhibit basal and estrogen-induced cell proliferation in the ZR-75-1 human breast cancer cell line. Breast Cancer Res. Treat..

[19] Ortmann J (2002). Testosterone and 5α-dihydrotestosterone inhibit in vitro growth of human breast cancer cell lines. Gynecol. Endocrinol..

[20] Lanzino M (2010). Inhibition of cyclin D1 expression by androgen receptor in breast cancer cells—identification of a novel androgen response element. Nucleic Acids Res..

[21] Gillett C (1996). Cyclin D1 and prognosis in human breast cancer. Int. J. Cancer.

[22] Zhou ZX, Lane MV, Kemppainen JA, French FS, Wilson EM (1995). Specificity of ligand-dependent androgen receptor stabilization: receptor domain interactions influence ligand dissociation and receptor stability. Mol. Endocrinol..

[23] Sonneveld, E., Jansen, H. J., Riteco, J. A., Brouwer, A. & van der Burg, B. J. T. S. Development of androgen-and estrogen-responsive bioassays, members of a panel of human cell line-based highly selective steroid-responsive bioassays. *Toxicol Sci.* **83**, 136–148 (2004). 10.1093/toxsci/kfi005 15483189

[24] Geisler J, Lønning PEJCCR (2005). Aromatase inhibition: translation into a successful therapeutic approach. Clin. Cancer Res..

[25] Byrns Michael C., Duan Ling, Lee Seon Hwa, Blair Ian A., Penning Trevor M. (2010). Aldo-keto reductase 1C3 expression in MCF-7 cells reveals roles in steroid hormone and prostaglandin metabolism that may explain its over-expression in breast cancer. The Journal of Steroid Biochemistry and Molecular Biology.

[26] PENNING Trevor M., BURCZYNSKI Michael E., JEZ Joseph M., HUNG Chien-Fu, LIN Hseuh-Kung, MA Haiching, MOORE Margaret, PALACKAL Nisha, RATNAM Kapila (2000). Human 3α-hydroxysteroid dehydrogenase isoforms (AKR1C1–AKR1C4) of the aldo-keto reductase superfamily: functional plasticity and tissue distribution reveals roles in the inactivation and formation of male and female sex hormones. Biochemical Journal.

[27] Giudici D (1988). 6-Methylenandrosta-1, 4-diene-3, 17-dione (FCE 24304): a new irreversible aromatase inhibitor. J. Steroid Biochem..

[28] Di Salle E (1990). 4-Aminoandrostenedione derivatives: a novel class of irreversible aromatase inhibitors. Comparison with FCE 24304 and 4-hydroxyandrostenedione. J. Steroid Biochem. Mol. Biol..

[29] Kandouz M (1999). Proapoptotic effects of antiestrogens, progestins and androgen in breast cancer cells. J. Steroid Biochem. Mol. Biol..

[30] Brodie AM, Schwarzel WC, Shaikh AA, Brodie HJ (1977). The Effect of an aromatase inhibitor, 4-Hydroxy-4-androstene-3, 17-dione, on estrogen-dependent processes in reproduction and Breast Cancer1. Endocrinology.

[31] Vaneecloo F, Piquet J, Van Ton J, Lejeune E, Jomin M (1989). Enlarged transfacial approach in the surgery of tumors of the ethmoid. Rev. Laryngol.-Otol.-Rhinol..

[32] Ando S (2002). Breast cancer: from estrogen to androgen receptor. Mol. Cell. Endocrinol..

[33] Greeve M, Allan R, Harvey J, Bentel J (2004). Inhibition of MCF-7 breast cancer cell proliferation by 5alpha-dihydrotestosterone; a role for p21 (Cip1/Waf1). J. Mol. Endocrinol..

[34] Macedo LF (2006). Role of androgens on MCF-7 breast cancer cell growth and on the inhibitory effect of letrozole. Cancer Res..

[35] CHOE MH (2018). Centrosome clustering is a tumor-selective target for the improvement of radiotherapy in breast cancer cells. Anticancer Res..

[36] Wu J-T, Han B-M, Yu S-Q, Wang H-P, Xia S-J (2010). Androgen receptor is a potential therapeutic target for bladder cancer. Urology.

[37] Morelli C (2004). Nuclear insulin receptor substrate 1 interacts with estrogen receptor α at ERE promoters. Oncogene.

[38] Yamasaki K (2003). OECD validation of the Hershberger assay in Japan: phase 2 dose response of methyltestosterone, vinclozolin, and p, p’-DDE. Environ. Health Perspect..

